# Extracellular Ribosomal RNA Acts Synergistically with Toll-like Receptor 2 Agonists to Promote Inflammation

**DOI:** 10.3390/cells11091440

**Published:** 2022-04-24

**Authors:** Karsten Grote, Marina Nicolai, Uwe Schubert, Bernhard Schieffer, Christian Troidl, Klaus T. Preissner, Stefan Bauer, Silvia Fischer

**Affiliations:** 1 Cardiology & Angiology, Medical School, Philipps-University, 35043 Marburg, Germany; grotek@staff.uni-marburg.de (K.G.); schieferb@staff.uni-marburg.de (B.S.); 2 Institute of Immunology, Medical School, Philipps-University, 35043 Marburg, Germany; nicolai5@staff.uni-marburg.de (M.N.); bauerst@staff.uni-marburg.de (S.B.); 3 Institute of Biochemistry, Medical School, Justus-Liebig-University, 35392 Giessen, Germany; uwe.schubert@biochemie.med.uni-giessen.de; 4 Medical Clinic I, Cardiology/Angiology, Campus Kerckhoff, Justus-Liebig-University, 61231 Bad Nauheim, Germany; christian.troidl@innere.med.uni-giessen.de; 5 Department Cardiology, Kerckhoff-Heart Research Institute, Medical School, Justus-Liebig-University, 35392 Giessen, Germany; klaus.t.preissner@biochemie.med.uni-giessen.de

**Keywords:** extracellular RNA, inflammation, cytokines, macrophages, endothelial cells, toll-like receptors

## Abstract

Self-extracellular RNA (eRNA), which is released under pathological conditions from damaged tissue, has recently been identified as a new alarmin and synergistic agent together with toll-like receptor (TLR)2 ligands to induce proinflammatory activities of immune cells. In this study, a detailed investigation of these interactions is reported. The macrophage cell line J774 A.1 or C57 BL/6 J wild-type mice were treated with 18S rRNA and different TLR2 agonists. Gene and protein expression of tumor necrosis factor *(Tnf)-α*; interleukin *(Il)-1β, Il-6*; or monocyte chemoattractant protein *(Mcp)-1* were analyzed and furthermore *in vitro* binding studies to TLR2 were performed. The TLR2/TLR6-agonist Pam_2_ CSK_4_ (Pam2) together with 18S rRNA significantly increased the mRNA expression of inflammatory genes and the release of TNF-α from macrophages in a TLR2- and nuclear factor kappa B (NF-κB)-dependent manner. The injection of 18S rRNA/Pam2 into mice increased the cytokine levels of TNF-α, IL-6, and MCP-1 in the peritoneal lavage. Mechanistically, 18S rRNA built complexes with Pam2 and thus enhanced the affinity of Pam2 to TLR2. These results indicate that the alarmin eRNA, mainly consisting of rRNA, sensitizes TLR2 to enhance the innate immune response under pathological conditions. Thus, rRNA might serve as a new target for the treatments of bacterial and viral infections.

## 1. Introduction

Toll-like receptors (TLRs) are the best-characterized members of the family of pattern recognition receptors (PRRs) on host cells in innate immunity and trigger inflammation, induced by pathogen-associated molecular patterns (PAMPs) of infectious microbes as well as damage-associated molecular patterns (DAMPs) as endogenous alarmins. On a structural basis, each TLR contains a variable number of extracellular leucine-rich-repeats (LRR), which are involved in ligand recognition [1], a transmembrane domain, and an intracellular tail containing the Toll/IL-1 receptor (TIR) domain [2]. In immune cells, TLR1, TLR2, TLR4, TLR5, and TLR6 are expressed on the cell surface and recognize mainly bacterial products such as lipopeptides, peptidoglycans, lipopolysaccharide (LPS), or flagellin, whereas endosomal TLRs, such as TLR3, TLR7, and TLR9 respond to nucleic acid structures, which are only accessible after uptake of these microbial products by host cells [3].

The recognition of DAMPs, released by dying or damaged cells under stress conditions such as ischemia/reperfusion or mechanical trauma, promotes sterile inflammation, which is important for restoring tissue homeostasis, tissue repair, and regeneration. On the other hand, DAMPs can also lead to the development of numerous inflammatory diseases or cancer [4]. DAMPs or alarmins include cytosolic, mitochondrial, or nuclear components such as heat shock proteins, high mobility group box 1 (HMGB1), histones, and self-nucleic acids (including nuclear DNA and several types of RNA, especially rRNA).

Self-extracellular RNA (eRNA) released from damaged tissue or cells was identified by our group as a new alarmin by contributing to disease progression in ischemic stroke, thrombosis, myocardial infarction, atherosclerosis, rheumatoid arthritis, and cancer [5,6,7,8,9,10,11,12].

eRNA is not only released by passive but also by active processes, which are dependent on an increase in the intracellular Ca^2+^ concentration leading to the release of microvesicle-associated eRNA [13,14]. Analysis of eRNA in cell supernatants or plasma samples by gel-electrophoresis revealed that rRNA is the main component of eRNA [13,15]. Accordingly, the amount of rRNA present in all eukaryotic cells was determined to be about 80–90% of the total cellular RNA [16]. In previous studies, eRNA was shown to induce prothrombotic, permeability-increasing, and inflammatory responses in immune and vascular cells [5,6,7,8,17]. Additionally, lower concentrations of eRNA can serve as a potent adjuvant, particularly for TLR2 ligands on macrophages, to increase their proinflammatory potential in a synergistic manner [14,18]. Moreover, eRNA can sensitize astrocytes, active players in cerebral innate immunity, towards exogenous and endogenous activators of inflammation (such as HMGB1) in a synergistic manner via TLR2-NF-κB-dependent signaling pathways [14].

The present study aimed to gain further insight into the mechanism of the synergistic action of eRNA and TLR2 agonists by using different TLR ligands, rRNA fragments, and by performing *in vitro* TLR2-binding studies.

## 2. Materials and Methods

### 2.1. Cell Culture

The monocyte/macrophage cell line J774 A.1 was grown in Dulbecco’s modified Eagle medium (DMEM) and Glutamax medium (Gibco, Darmstadt, Germany) containing 10% fetal calf serum (FCS, Gibco) and 1% penicillin/streptomycin (Sigma-Aldrich, Munich, Germany). The endothelial cell line MyEND, showing typical endothelial properties, was grown in DMEM with 10% FCS and 1% penicillin/streptomycin, as recently described [19].

The following agents were used for cell treatments: Pam_2_ CSK_4_, Pam_3_ CSK_4_ (Pam3), MAb-mTLR2, and mouse IgG from invivoGen (Toulouse, France); PD98059, SB203580, and SP600125 from Calbiochem (Merk, Darmstadt, Germany); and Bay 11–7082 from Enzo Life Sciences (Lörrach, Germany). The macrophage-activating lipopeptide of 2 kDa (MALP-2) was synthesized and purified as described before [20]. Before stimulation, cells were washed once with phosphate-buffered saline (PBS, Sigma-Aldrich) and incubated for the indicated periods in FCS-free cell culture medium containing the different agents at the indicated concentrations. The stimulation of cells with eRNA/Pam_2_ CSK_4_, 18S rRNA/ Pam_2_ CSK_4_, 18S rRNA/Pam_3_ CSK_4_, and 18S rRNA/MALP-2 mixtures was performed after preincubating both agents in double-distilled water for 30 min at 37 °C.

### 2.2. Mice

Mice were housed in individually ventilated cages (IVC) under specific pathogen-free (SPF) conditions in the local animal facility. Eight to ten-weeks-old male and female C57 BL/6 J wild-type mice were intraperitoneally injected with 10 ng Pam_2_ CSK_4_, 1 µg 18S rRNA, or a combination of both in 250 µL PBS. The mixture was prepared as for the cell culture experiments. After 4 h, peritoneal lavage with 3 mL of PBS was performed and the blood was collected. All experiments were approved by the governmental animal ethics committee (G13/2018) and conformed to the guidelines from directive 2010/63/EU of the European Parliament.

### 2.3. Isolation and Quantification of RNA

eRNA, which was used to stimulate cells, was isolated from confluent cultures of mouse fibroblasts using a commercially available kit (Peqlab, Erlangen, Germany) and extracted additionally two times with Trizol according to the manufacturer’s instructions (kit from Thermo Fisher Scientific, Waltham, MA, USA). eRNA from cell supernatants was isolated as previously described [18]. Briefly, cell supernatants were first centrifuged for 5 min at 200× *g* to remove cells and cell debris. To prevent degradation of RNA, RNase inhibitor (4 U/mL, RNasin, Invitrogen) was added and samples were concentrated using centricon tubes (cut off 10 kDa; Millipore, Burlington, MA, USA) that were centrifuged at 3400× *g* for 12 min at 4 °C and subsequently washed with autoclaved sterile water. The same amounts of lysis buffer (peqGOLD total RNA kit from Peqlab) were added to the concentrated cell supernatants and RNA was isolated in accordance with the instructions of the manufacturer. RNA from lysates or cell supernatants was quantified using the NanoDrop 2000 (Thermo Fisher Scientific) and the quality of RNA was confirmed by electrophoresis on 1% agarose gels followed by ethidium bromide staining or by using the Agilent 2100 bioanalyzer and the Agilent RNA 6000 Nano Kit (Agilent Technologies, Konstanz, Germany), which demonstrated that the major components of isolated RNAs were 28S and 18S rRNA. Furthermore, the purity of RNA was confirmed by performing the endotoxin test using the Pierce^TM^ LAL chromogenic endotoxin quantification kit (Thermo Fisher Scientific).

### 2.4. In Vitro Transcription of Human 18S rRNA

Human 18S rRNA (NR_145820.1) was amplified from genomic HEK293 DNA using the primer pair 5′-TAC CTG GTT GAT CCT GCC AGT AGC-3′ and 5′-TAA TGA TCC TTC CGC AGG TTC ACC TAC-3′ and cloned into pGEM^®^-T Easy Vector (Promega, Mannheim, Germany). Fragments of 18S rRNA (18S-1, 18S-2 and 18S-3) were amplified from full-length human 18S rRNA plasmid using the following primers: 18S-1: 5′-TAC CTG GTT GAT CCT GCC AG-3′ and 5′-GCC GTC CCT CTT AAT CAT GG-3′, 18S-2: 5′-CGG GGG CAT TCG TAT TGC GC-3′ and 5′-TAA TGA TCC TTC CGC AGG TTC-3′, 18S-3: 5′-GAC CCG CCG GGC AGC TTC CG-3′, and 5′-CTG CCG GCG TAG GGT AGG CAC-3′. For in-vitro transcription, a pGEM plasmid containing human 18S rRNA was linearized with Pvu II and purified with GeneJet PCR purification kit (ThermoFisher, Germany) according to the manufacturer’s recommendation or alternatively extracted with phenol/chloroform, precipitated, and solubilized in reaction buffer. RNA was produced *in vitro* using T7-ScribeTM Standard RNA IVT Kit, CELLSCRIPTTM according to the manufacturer’s recommendation. After synthesis and removal of DNA by DNase I digestion, the RNA mixture was desalted with Micro Bio-Spin™ Chromatography Columns (BIO RAD).

### 2.5. Electrophoretic Mobility Shift Assay (EMSA)

18S rRNA alone or preincubated with different concentrations of Pam2 for 30 min at 37 °C in the absence or presence of 1% SDS were separated on 0.7% agarose gels.

### 2.6. TLR2 Fusion Protein

The extracellular domain of murine toll-like receptor 2 (aa 1–587) was fused to the human IgG1-Fc protein and expressed in HEK293 cells [21]. Following the concentration of the 5-L cell supernatant with a Vivaflow 200 (Sartorius) ultrafiltration cassette (50 kDa molecular weight cut-off), the TLR2 fusion protein was purified by protein A affinity chromatography, and the purity was verified by SDS-polyacrylamide gel electrophoresis (PAGE) and Coomassie staining.

### 2.7. TLR2 Binding Assay

Maxisorp NUNC-immuno plates were coated with streptavidin (from Streptomyces avdinii, Sigma) at 1 µg/well in PBS and incubated at 4 °C overnight. All incubations were performed in a humid chamber. To avoid unspecific binding, the plates were blocked with PBS containing 1% BSA (Sigma-Aldrich) for 1 h at 37 °C. After the blocking procedure, the plates were washed three times with pre-warmed PBS.

To investigate the interactions of Pam2 with human 18S rRNA and TLR2 fusion protein, Pam2-Biotin (Pam2-Biotin-Aca-Aca-NH2, Genaxxon bioscience) was preincubated with human 18S rRNA in ultra-pure water (10 µL) for 30 min at 37 °C. Subsequently, TLR2 fusion protein and medium were added (pure Opti-MEMTM medium, Gibco) for an additional 30 min at 37 °C. This mixture was added to the streptavidin-coated plates (50 µL/well) and incubated for 15 min at 37 °C, followed by three washing steps with PBS.

For the detection of TLR2, each well was incubated with an anti-human IgG-peroxidase conjugated antibody (1:1000, Dako) for 1 h at 37 °C. After the last washing step, a substrate buffer with 20 mg o-phenylenediamine dihydrochloride (OPD, Sigma) and 30% of H_2_ O_2_ was applied to the wells (50 µL) and incubated for approximately 20 min at room temperature in the dark. The reaction was stopped by adding 25 µL/well 2 M H_2_ SO_4_, and absorption was measured with a photometer at 450–650 nm

### 2.8. Quantitative Real-Time PCR

Following the treatment of J774 A.1 or MyEND cells with various agonists as indicated in the legends of the corresponding figures, cells were washed twice with PBS, lysed, and RNA was isolated with the GenElute Mammalian Total RNA Miniprep Kit (Sigma). For real-time PCR analysis, 1 µg of RNA was reverse-transcribed using the High-Capacity cDNA Reverse Transcription Kit (Applied Biosystems, Carlsbad, CA, USA), and DNA amplification was performed with a StepOne Plus cycler (Applied Biosystems) and analyzed with the StepOne^TM^ software (v2.3) in a reaction volume of 10 µL using the SensiMix Sybr Kit (Bioline, Luckenwalde, Germany) with 50 pmol of each primer. To avoid amplification of the genomic DNA, primers were designed to span exon–exon junctions. The real-time PCR was performed under the following conditions: an initial denaturation step at 95 °C for 8.5 min followed by 45 cycles, consisting of denaturation (95 °C, 30 s), annealing (60 °C, 30 s) and elongation (72 °C, 30 s). Melt curve analysis was performed to control the specific amplification. Results were normalized to the expression levels (E) of actin and expressed as the ratio of E(target)/E(Actin). The following mouse primers were used: *Tnf-α* forward 5′-ACT GAA CTT CGG GGT GAT CG-3′,*Tnf-α* reverse 5′-TGG TTT GTG AGT GTG AGG GTC-3′, *Il-1β* forward 5′-GGA TGA GGA CAT GAG CAC CT-3′, *Il-1β* reverse 5′-GGA GCC TGT AGT GCA GTT GT-3′, *Il-6* forward 5′-CTC TGC AAG AGA CTT CCA TCC A-3′, *Il-6* reverse 5′-TTG TGA AGT AGG GAA GGCCG-3′,*Mcp-1* forward 5′-AAG CTG TAGTTT TTG TCA CCA AGC-3′, *Mcp-1* reverse 5′-GAC CTT AGG GCA GAT GCA GTT-3′, *Tlr2* forward 5′-TCT TGT TTC TGA GTG TAG GGG C-3′, *Tlr2* reverse 5′-CAT CCT CTG AGA TTT GAC GCT TTG-3′, *Tlr6* forward 5′-TGA ATG ATG AAA ACT GTC AAA GGT TAA-3′, *Tlr6* reverse 5′-GGG TCA CAT TCA ATA AGG TTG GA-3′, *actin* forward 5′-CGC GAG CAC AGC TTC TTT G-3′, and *actin* reverse 5′-CGT CAT CCA TGG CGA ACT GG-3′.

### 2.9. Enzyme-Linked Immunosorbent Assay (ELISA)

Supernatants from J774 A.1 cells, MyEND cells, as well as samples from peritoneal lavage were analyzed by ELISA. ELISAs for TNF-α (detection limit = 8 pg/mL), Il-1β (detection limit = 8 pg/mL), IL-6 (detection limit = 4 pg/mL), and MCP-1 (detection limit = 15 pg/m) were performed using the commercially available kit from eBioscience (Frankfurt, Germany).

### 2.10. Statistical Analysis

All data were represented as means ± SEM. Two-tailed unpaired Student t-test was used to compare two independent groups; one-way ANOVA followed by Fisher´s LSD post hoc test was used when more than two groups with one independent variable were compared and two-way ANOVA followed by Fisher´s LSD post hoc or Tukey’s multiple comparison tests were used when more than two groups with two independent variables were compared (GraphPad Prism, version 7.0; GraphPad Software, La Jolla, CA). A value of P < 0.05 was considered statistically significant. The numbers of independent experiments were indicated in the respective figure legends.

## 3. Results

### 3.1. Synergistic Activity of 18S rRNA and Pam_2_ CSK_4_

**Figure 1:**
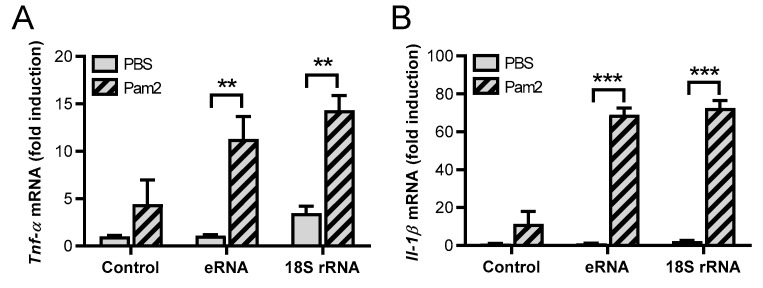

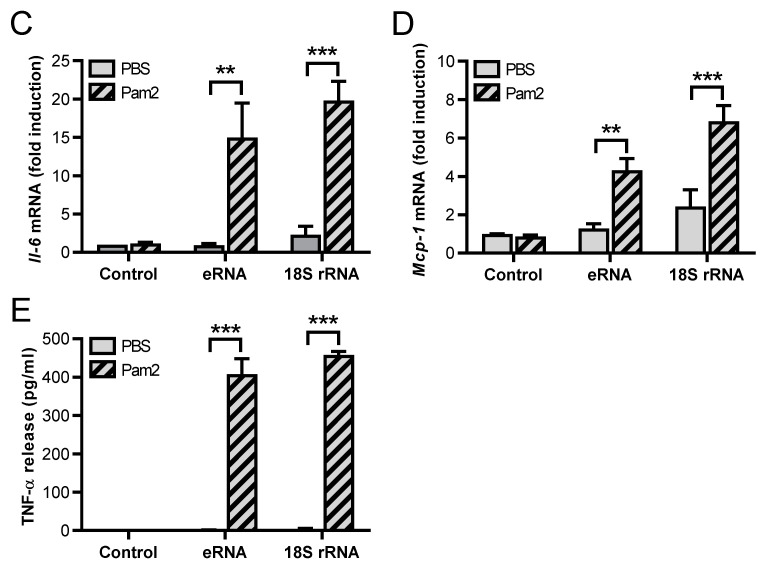
Synergistic activity of 18S rRNA and Pam_2_ CSK_4_. Macrophages (J774 A.1 cells) were treated for 2 h with Pam_2_ CSK_4_ (Pam2, 0.1 ng/mL) and either self-extracellular RNA (eRNA, 1 µg/mL) or 18S rRNA (1 µg/mL) alone or with the preincubated mixture of Pam2 with either eRNA or 18S rRNA. PBS-treated cells without any additives served as control. Real-time PCR was used to determine transcript levels of *Tnf-α* (**A**), *Il-1β* (**B**), *Il-6* (**C**), or *Mcp-1* (**D**). TNF-α protein levels in cellular supernatants were quantified by ELISA (**E**). Values are expressed as mean ± SEM; *N* = 3; ** *p* < 0.01, *** *p* < 0.001 between indicated groups.

In previous studies, we demonstrated that eRNA synergistically enhanced TLR2 ligand-induced expression of cytokines and their secretion from murine macrophages (differentiated from bone marrow-derived stem cells) by shifting the dose-response curve (and the IC50 value) for the TLR2 ligand Pam2 to much lower concentrations [18]. As eRNA appears to be heterogeneous with regard to the composition of RNA and mainly consists of rRNA, we wanted to study whether purified full-length 18S rRNA, synthesized by *in vitro* transcription, could duplicate the observed results using the macrophage cell line J774 A.1. The observed data were initially compared with those of eRNA isolated from mouse fibroblasts. The preincubated mixtures consisting of low concentrations of Pam2 (0.1 ng/mL) and either eRNA or 18S rRNA significantly increased the mRNA expression of inflammatory cytokines such as *Tnf-α*, *Il-1β, Il-6*, or *Mcp-1* (Figure 1A–D), as well as the protein release of TNF-α from macrophages as compared to each of the RNA-forms alone as agonists (Figure 1E). In all experiments, early time points were used to avoid secondary effects such as autocrine effects of TNF-α [18]. Furthermore, 18S rRNA/Pam2 had a slightly higher (although not significant) potency compared to eRNA/Pam2 in mediating cytokine expression. 18S rRNA, eRNA, and Pam2 at low concentrations alone showed no or only minor effects on the cytokine expression without any significant differences between them (Figure 1A–E).

### 3.2. Signaling Pathways Involved in 18S rRNA/Pam_2_ CSK_4_-Induced Activities

While primary macrophages were previously used to study the activities of Pam2/eRNA, the involvement of TLR2 in the induction of gene expression by Pam2 and 18S rRNA could now be confirmed with the macrophage cell line J774 A.1. 18S rRNA/Pam2-induced cytokine induction was inhibited by a neutralizing antibody against TLR2, whereas the corresponding control IgG did not show any effect (Figure S1A–D). In accordance with the eRNA/Pam2-induced signaling pathways identified in primary macrophages, the blockade of the NF-κB pathway by Bay completely abolished the mRNA expression of *Tnf-α, Il-1β,* and *Il-6,* as well as the release of TNF-α in J774 A.1 cells. Also, the activation of the mitogen-activated protein (MAP)-kinase p38 was involved in 18S rRNA/Pam2-mediated mRNA expression of *Il-1β*, as shown in experiments using the p38 MAP-kinase inhibitor SB203580 (Figure S1A–D). Likewise, the release of TNF-α—but not the mRNA expression of *Tnf-*α—was dependent on MAP-kinase p38-signaling (Figure S1A,D). The activation of MAP-kinase 42/44 as well as of c-Jun N-terminal kinase (JNK) was not involved in inflammatory activities of 18S rRNA/Pam2, as cytokine induction was not blocked by MAP-kinase 42/44 pathway inhibitor PD98059 or the JNK inhibitor SP600125, respectively (Figure S1A–D). These results indicate that J774 A.1 cells proved to be a suitable cell line for further studies since the inflammatory potential of 18S rRNA/Pam2 in the J774 A.1 macrophage cell line appeared to be transmitted by the same mechanisms as in primary macrophages. Therefore, all the following experiments were performed using the macrophage cell line.

**Figure 2:**
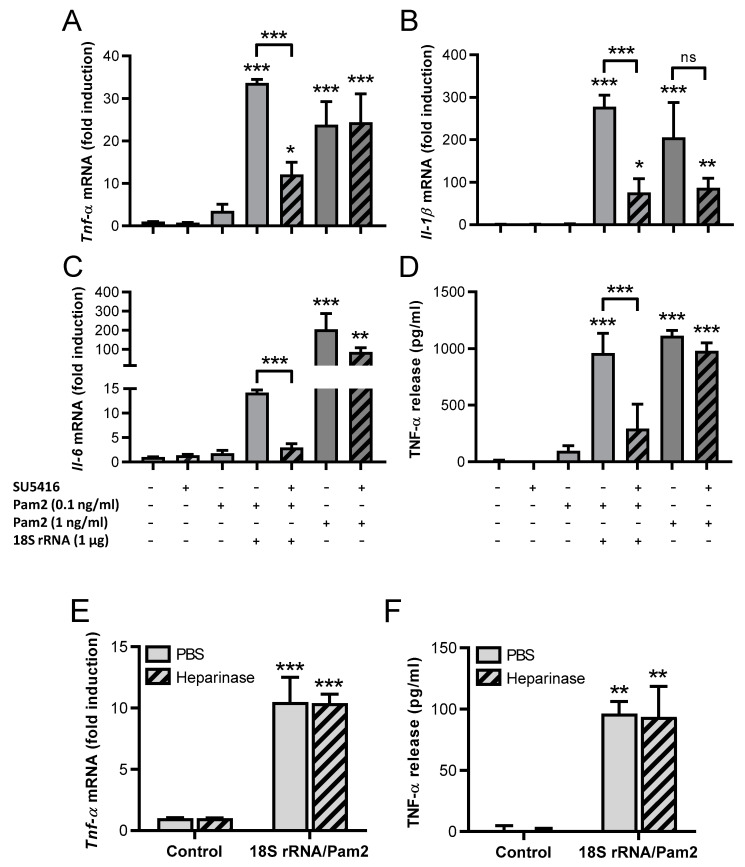
Synergistic activity of 18S rRNA and Pam_2_ CSK_4_ after blocking VEGF-R2 activation and heparinase treatment. (**A**–**D**): Macrophages (J774 A.1 cells) were treated for 2 h with a preincubated mixture of Pam_2_ CSK_4_ (Pam2, 0.1 ng/mL) and 18S rRNA or Pam2 alone (1 ng/mL), both without or after pretreatment of cells with SU5416 (10 mM) and additionally with Pam2 alone (0.1 ng/mL). Real-time PCR was used to determine transcript levels of *Tnf-α* (**A**), *Il-1β* (**B**), or *Il-6* (**C**). TNF-α protein levels in cellular supernatants were quantified by ELISA (**D**). (**E**,**F**) Macrophages (J774 A.1) were pretreated for 1 h with heparinase (50 mU/mL), stimulated with a preincubated mixture of Pam_2_ CSK_4_ (Pam2, 0.1 ng/mL) and 18S rRNA (1 μg/mL) for 2 h, and *Tnf-α* expression was analyzed by real-time PCR, (**E**) and the release of TNF-α protein levels was quantified by ELISA (**F**). PBS-treated cells without any additives served as control. Values are expressed as mean ± SEM; N = 3–6; * *p* < 0.05, ** *p* < 0.01, *** *p* < 0.001 versus control value or between indicated groups.

Previous investigations of our group with endothelial cells demonstrated that eRNA acts via the activation of vascular endothelial growth factor (VEGF) receptor 2 (VEGF-R2) by increasing the binding of VEGF to its receptor [22]. Likewise, in J774 A.1 cells, the potent and selective VEGF-R2 tyrosine kinase inhibitor SU5416 [23] significantly decreased 18S rRNA/Pam2-induced expression of *Tnf-α, Il-1β,* and *Il-6*, as well as the release of TNF-α (Figure 2A–D). In contrast, the cytokine expression induced by higher concentrations of Pam2 was not decreased by SU5416. These data indicate that VEGF-R2 is involved in the synergistic activities of 18S rRNA/Pam2 but not in TLR2 activation induced by higher concentrations of active Pam2 alone.

To investigate if regulatory proteins such as growth factors, which are known to bind to cell membrane-bound heparan sulfate proteoglycans, are involved in the 18S rRNA/Pam2-induced effects, cells were pretreated with heparinase to remove the glycosaminoglycans. Yet, pretreated macrophages did not change the expression and release of TNF-α in response to the 18S rRNA/Pam2 agonist (Figure 2E,F), indicating that heparan sulfate proteoglycans are not involved in cellular activation.

### 3.3. Synergistic Effects of 18S rRNA with Other TLR Ligands

**Figure 3:**
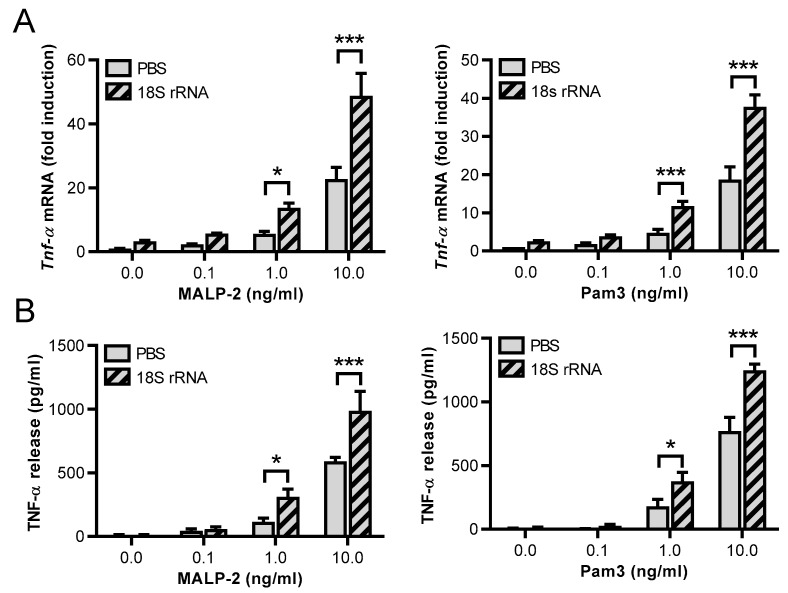

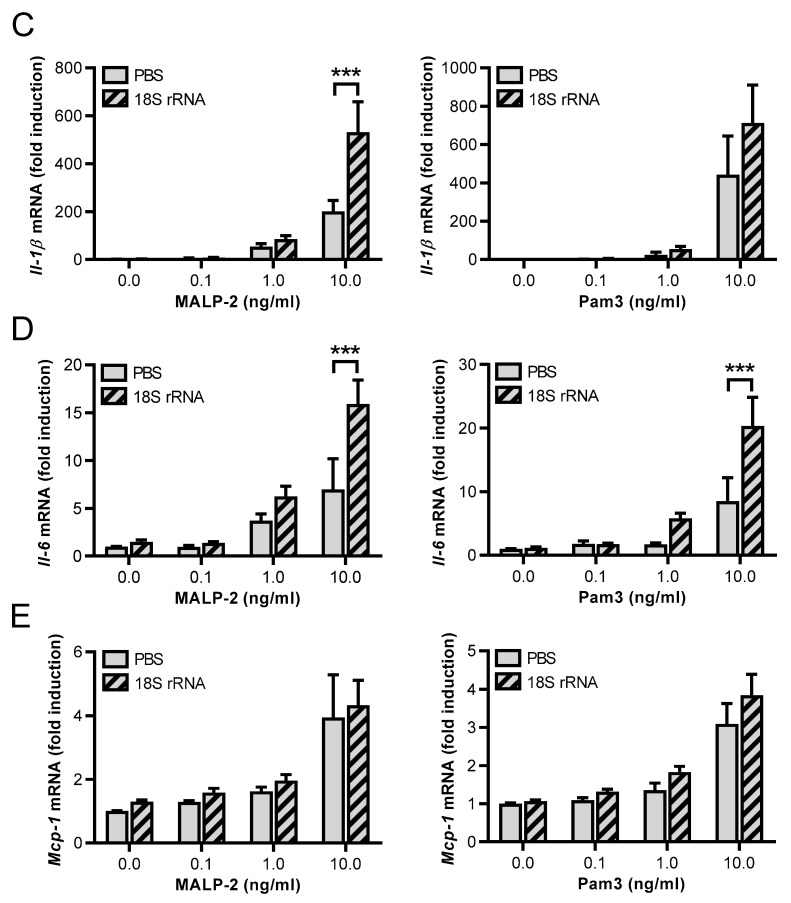
Synergistic activity of 18S rRNA together with the TLR ligands MALP-2 and Pam_3_ CSK_4_. Macrophages (J774 A.1 cells) were treated for 2 h with different concentrations of MALP-2, Pam3, or 18S rRNA (1 µg/mL) alone or with a preincubated mixture of different concentrations of MALP-2 or Pam3 together with 18S rRNA. PBS-treated cells without any additives served as control. Real-time PCR was used to determine transcript levels of *Tnf-α* (**A**), *Il-1β* (**C**), *Il-6* (**D**), or *Mcp-1* (**E**). TNF-α protein levels in cellular supernatants were quantified by ELISA (**B**). Values are expressed as mean ± SEM; N = 3–10; * *p* < 0.05, *** *p* < 0.001 between indicated groups.

To further analyze the availability and specificity of 18S rRNA for other TLR ligands, MALP-2 (another TLR2/TLR6 agonist) and the TLR2/TLR1 ligand Pam3 were used. Alone, both agonists induced *Tnf-α* mRNA expression in a concentration-dependent manner in J774 A.1 cells (Figure S2). To investigate potential synergistic effects with 18S rRNA, MALP-2 and Pam3 were used at low concentrations from 0.1–10 ng/mL, which per se had no or only a moderate influence on *Tnf*-α mRNA expression. The presence of 18S rRNA (1 µg/mL) synergistically increased MALP-2- and Pam3-induced *Tnf*-α mRNA expression as well as TNF-α release (Figure 3A,B). Of note, compared to Pam2 and 18S rRNA, higher concentrations of each respective TLR2 ligand were required (1 ng/mL for MALP-2 and Pam3 vs. 0.1 ng/mL for Pam2) to observe the described cell activation. The same results were obtained for *Il-6*, whereby *Il-1β* gene expression was only significantly increased by 18S rRNA/MALP-2 (Figure 3C,D). Also, the *Mcp-1* transcript level was not further elevated (Figure 3E). 18S rRNA alone (left pair of bars without MALP-2 or Pam3) was unable to induce cytokine expression.

### 3.4. Detection of 18S rRNA/Pam2 Complexes and Influence of 18S rRNA on Pam2 Binding to TLR2

**Figure 4:**
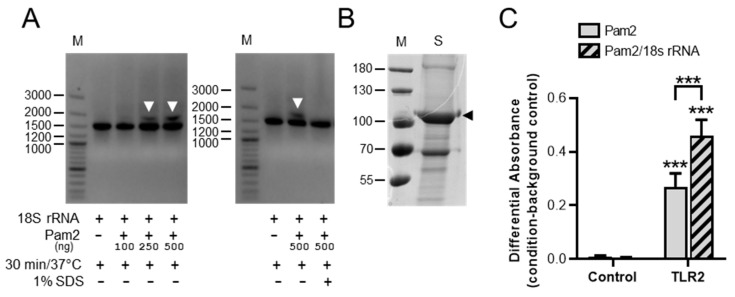
Detection of 18S rRNA/Pam2 complexes and binding assay of Pam2 to TLR2. 18S rRNA alone or preincubated with different concentrations of Pam2 for 30 min at 37 °C in the absence or presence of 1% SDS were separated on 0.7% agarose gels. Arrowheads indicate 18S rRNA/Pam2 complex, M = DNA marker (size in bp) (**A**). SDS-PAGE and Coomassie staining of the extracellular domain of TLR2 fused to IgG1-Fc protein, expressed in HEK293 and purified from cell supernatants, was performed. Arrowhead indicates TLR2-Fc, M = protein marker (size in kDa), S = purified and concentrated supernatant from HEK293 cells (**B**). Pam2-Biotin (7.5 ng/mL) was preincubated for 30 min in the absence or presence of 18S rRNA (0.1 μg/mL) and subsequently with the TLR2 fusion protein (10 µg/mL) (**C**). The binding of Pam2-Biotin to TLR2 was measured after binding to streptavidin and detection of bound TLR2 by IgG-peroxidase-conjugated antibody. After adding the peroxidase substrate, the absorbance of the product was measured at 450–650 nm. The mean values are presented as mean ± SEM; N = 3–4; *** *p* < 0.001 versus corresponding control values or between indicated groups.

To investigate a possible interaction of 18S rRNA with Pam2, we performed electrophoretic mobility shift assays. Preincubation of 18S rRNA with increasing concentrations of Pam2 for 30 min at 37 °C resulted in a dose-dependent shift of a higher molecular band in the gel, indicating the formation of 18S rRNA/Pam2 complexes. The formation of this complex was prevented by the addition of detergent (1% SDS) during preincubation (Figure 4A).

To investigate whether the binding affinity of Pam2 to TLR2 is influenced by the presence of 18S rRNA, *in vitro* binding assays were performed. For these studies, the extracellular domain of murine TLR2 was fused to the IgG1-Fc protein and expressed in HEK293 cells. Following purification of the fusion protein from cell supernatants, the purity of the construct was verified by SDS-gel electrophoresis and used for interaction studies (Figure 4B). The binding of Pam2 to TLR2 fusion protein was significantly increased in the presence of 18S rRNA, which corresponds with the high inflammatory potency of the 18S rRNA/Pam2 complex in the previous cell assays (Figure 4C).

### 3.5. Synergistic Activities of Different 18S rRNA Fragments Together with Pam_2_ CSK_4_

**Figure 5:**
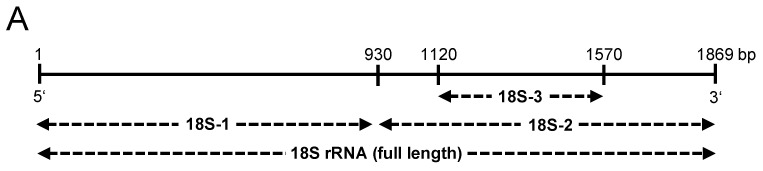

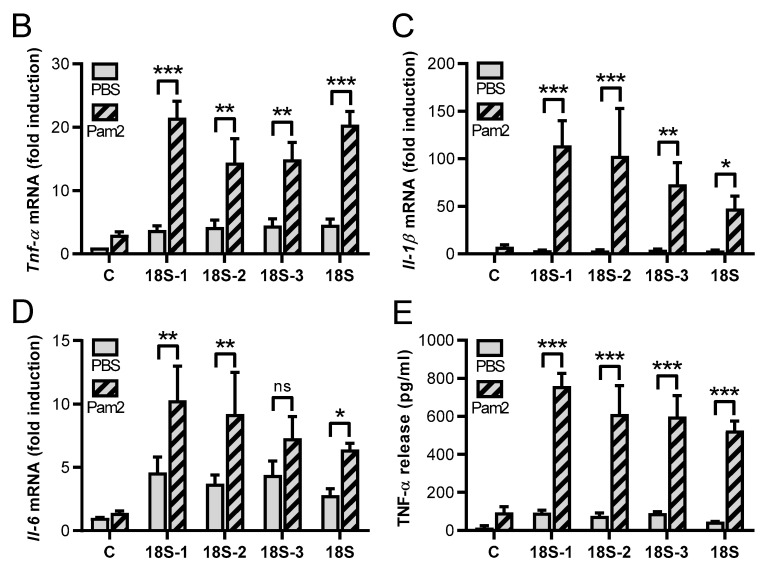
Synergistic activities of 18S rRNA fragments with Pam_2_ CSK_4_. The 18S rRNA fragments of different sizes were generated by *in vitro* transcription (**A**). Macrophages (J774 A.1 cells) were treated for 2 h with Pam_2_ CSK_4_ (Pam2, 0.1 ng/mL), 18S rRNA (1 µg/mL), or 18S rRNA fragments (18S-1, 18S-2, 18S-3; each 1 µg/mL) alone or with preformed complexes together with Pam2 each. PBS-treated cells without any additives served as control. Real-time PCR was used to determine transcript levels of *Tnf-α* (**B**), *Il-1β* (**C**), or *Il-6* (**D**). TNF-α protein levels in cellular supernatants were quantified by ELISA (**E**). Values are expressed as mean ± SEM; N = 6–12; * *p* < 0.05, ** *p* < 0.01, *** *p* < 0.001 between indicated groups.

In order to investigate specific regions of 18S rRNA that might be responsible for the observed synergistic effects with the indicated PAMPs, different parts of the 18S rRNA were cloned. Afterwards, the respective RNA fragments of different sizes were *in vitro* transcribed (18S-1 = 5′-fragment, 18S-2 = 3′-fragment, 18S-3 = partial 3′-fragment, 18S = full-length) (Figure 5A). Following the stimulation of J774 A.1 cells with Pam2 in the presence of such fragments in comparison to the full-length 18S rRNA revealed that the different fragments were quite similar in their synergistic potential. However, the 18S rRNA fragment 18S-1 transcribed from the initial 5′-region of 18S rRNA (1–930 bp) increased *Tnf-α*, *Il-1β*, or *Il-6* mRNA expression, as well as TNF-α released the most, even though these differences were not significant compared to the other fragments and the full-length 18S rRNA (Figure 5B–E).

### 3.6. Synergistic Effects of 18S rRNA and Pam_2_ CSK_4_ In Vivo

**Figure 6:**
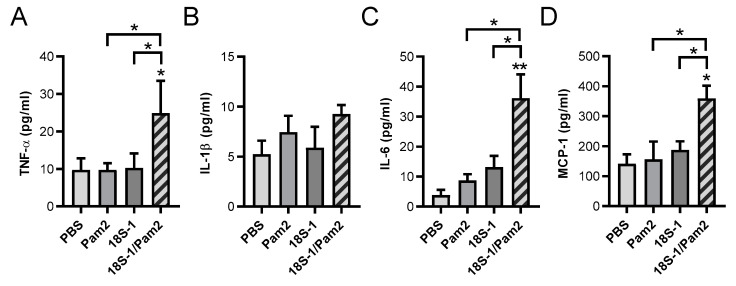
Synergistic activity of 18S rRNA and Pam_2_ CSK_4_ *in vivo*. C57 BL/6 J mice were intraperitoneally injected with either 10 ng Pam_2_ CSK_4_ (Pam2) or 1 µg 18S-1 rRNA alone or with preformed complexes of both in 250 µL PBS. PBS injection alone was used as sham control (PBS). After 4 h, peritoneal lavage was collected and TNF-α (**A**), IL-1β (**B**), IL-6 (**C**), and MCP-1 (**D**) protein levels were quantified by ELISA. Values are expressed as mean ± SEM; N = 4–9; * *p* < 0.05, ** *p* < 0.01 versus control value (PBS) or between indicated groups.

To evaluate the possible synergistic effects of 18S rRNA and Pam2 *in vivo*, the 18S rRNA fragment 18S-1 (1 µg) preincubated with Pam2 (10 ng) was intraperitoneally injected into C57 BL/6 J mice. 18S-1/Pam2 was found to significantly increase the protein levels of TNF-α, IL-6, and MCP-1, but not of IL-1β (at the detection limit of the ELISA) in the peritoneal lavage, whereas 18S-1 or Pam2 alone had no effect at these concentrations (Figure 6A–D).

### 3.7. Lack of Synergism between 18S rRNA and Pam_2_ CSK_4_ on Cytokine Induction in Endothelial Cells

**Figure 7:**
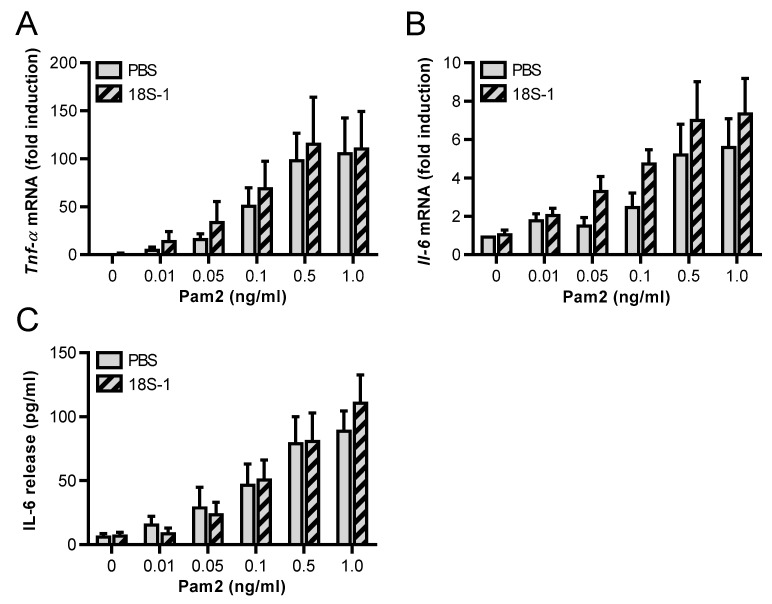
Lack of synergistic activity of 18S rRNA and Pam_2_ CSK_4_ in endothelial cells. Endothelial cells (MyEND cells) were treated for 3 h with different concentrations of Pam_2_ CSK_4_ (Pam2) and 18S rRNA (1 µg/mL) alone or with preformed complexes of different concentrations of Pam2 together with 18S rRNA. PBS-treated cells without any additives served as control. *Tnf-α* (**A**) and *Il-6* (**B**) mRNA levels were quantified by real-time PCR. Release of IL-6 (**C**) protein in cellular supernatants was quantified by ELISA. Values are expressed as mean ± SEM; N = 3–8.

In addition to the macrophage-like J774 A.1 cell line, the potential synergistic effects of 18S rRNA and Pam2 were tested on endothelial cells, which likewise express TLRs for pathogen recognition and immune defense. To this end, the endothelial MyEND cell line was used, which was recently characterized with regard to its endothelial-specific properties and the expression of *Tlr2* and *Tlr6* [19]. Following 3 h of stimulation, Pam2 alone increased the mRNA levels of *Tnf-α* and *Il-6,* as well as the protein secretion of IL-6 in a dose-dependent manner, being significant at ≥10 ng/mL Pam2. Contrary to J774 A.1 cells, TNF-α protein was not detectable in MyEND cells (Figure S3A–D), indicating a different posttranscriptional regulation of the mRNA or a different proteolytic activation of the protein in these cell types. As opposed to J774 A.1 cells, inactive low concentrations of Pam2 (up to 1 ng/mL) in the presence of 18S rRNA (1 µg/mL) were ineffective to exhibit any synergistic effects on the mRNA expression levels of *Tnf-α* or *Il-6* as well as on IL-6 protein secretion in MyEND cells (Figure 7A–C). As in J774 A.1 cells, 18S rRNA alone (left pair of bars without Pam2) was unable to induce cytokine expression in MyEND cells as well.

**Figure 8:**
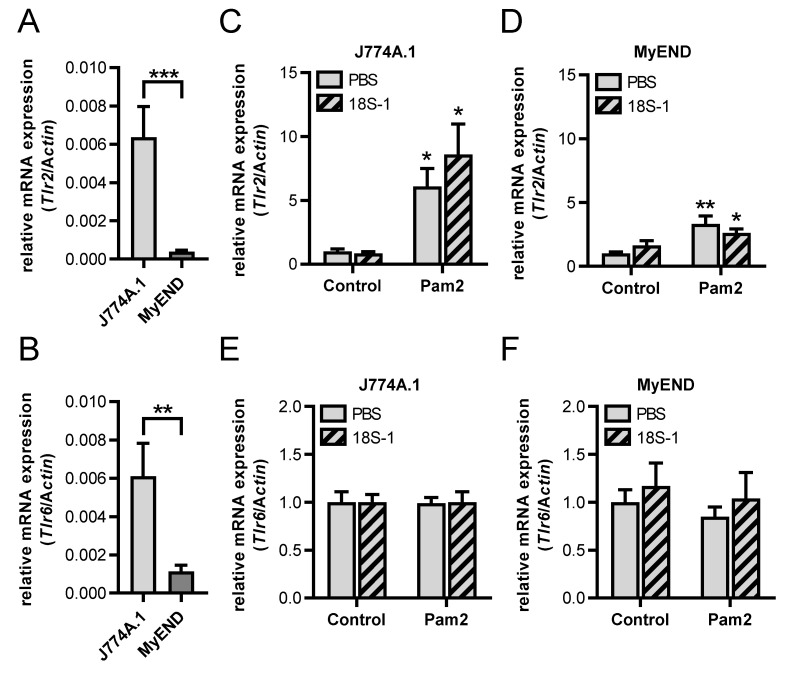
Influence of 18S rRNA on Pam_2_ CSK_4_-dependent *Tlr2* and *Tlr6* expression. *Tlr2* (**A**) and *Tlr6* (**B**) mRNA levels under basal conditions were quantified by real-time PCR in macrophages (J774 A.1 cells) and endothelial cells (MyEND cells). MyEND and J774 A.1 cells were treated for 3 h with 18S rRNA (1 µg/mL) alone or with preformed complexes of Pam_2_ CSK_4_ (Pam2, 100 pg/mL). PBS-treated cells without any additives served as control. *Tlr2* (**C**,**D**) and *Tlr6* (**E**,**F**) mRNA levels were quantified by real-time PCR. Values are expressed as mean ± SEM; N = 4–8; * *p* < 0.05, ** *p* < 0.01, *** *p* < 0.001 versus control value or between indicated groups.

These differences between macrophages and endothelial cells with regard to the synergistic activities of 18S rRNA/Pam2 on cytokine expression could be due to different expression levels or a different regulation pattern of the Pam2 receptors, TLR2 and TLR6, in these cell types. In fact, *Tlr2* and *Tlr6* expression was significantly lower expressed in MyEND cells compared to J774 A.1 cells (Figure 8A,B). Furthermore, Pam2-increased Tlr2 expression in macrophages was considerably more effective than in endothelial cells. However, the presence of 18S rRNA did not show any significant increase of Pam2-induced *Tlr2* mRNA expression in both cell types (Figure 8C,D). The expression of *Tlr6* mRNA was not changed by Pam2 alone or in the presence of 18S rRNA in macrophages or endothelial cells (Figure 8E,F).

## 4. Discussion

To study the established synergistic activities of eRNA with TLR2 ligands in more detail, in-vitro-transcribed 18S rRNA was used, which increased the expression of inflammatory genes such as *Tnf-α*, *Il-1β, Il-6*, and *Mcp-1,* as well as the release of TNF-α from the macrophage cell line J774 A.1 in a TLR2-dependent manner. Thus, we confirmed our previous results obtained with eRNA and primary bone marrow-derived macrophages and could further document that mainly rRNA is responsible for the described activities of eRNA [18].

Except for TLR3, most TLRs (including TLR1, TLR2, TLR4, TLR5, TLR7, TLR8, and TLR9) use the intracellular adaptor proteins myeloid differentiation primary response 88 (MyD88) and IL-1 receptor-associated kinases (IRAK-4 and -1) via their death domain interactions to activate the canonical NF-κB and MAP-kinase pathways, leading to the generation of proinflammatory cytokines such as TNF-α, IL-1β, or IL-6 [24,25,26]. According to our previous results, the signaling pathways induced by 18S rRNA/Pam2 also involved the activation of the NF-κB- and of MAP-kinase 38-dependent pathways [18,27].

Unlike other TLRs, which are functionally active as homodimers, TLR2 is known to exist as a heterodimer together with TLR1 or TLR6, respectively [28], to attain specificity for different lipopeptide ligands. The dimers utilize two TIR domain-containing adaptor proteins, MyD88 and TIR domain-containing adaptor protein (TIRAP), and subsequently activate NF-κB- and MAP-kinase-dependent signaling pathways [28]. In this regard, Pam2 is known to bind and activate TLR2/TLR6 dimers [29]. However, Pam2 is also active as an agonist in TLR6-deficient cells [30,31], indicating that TLR2 alone may also function as a Pam2 receptor. The synergistic activity of 18S rRNA on Pam2-induced, TLR2-dependent cytokine upregulation seems to involve the activation of VEGF-R2 as well because the overall cell activation was significantly decreased by SU5416 as a specific inhibitor of the tyrosine phosphorylation of VEGF-R2 [23]. Since the induced cytokine expression after stimulation of TLR2 by higher concentrations of Pam2 was unaffected by SU5416, a new coreceptor function of VEGF-R2 in the presence of low, by itself ineffective concentrations of Pam2 together with 18S rRNA is proposed.

It is already known that the TLRs require coreceptors. For example, the TLR2 heterodimer TLR2/TLR1 needs the coreceptor CD14, and the heterodimer TLR2/TLR6 additionally requires CD36, which both were supposed to act as an intermediate complex facilitating the loading of ligands to both TLR2 heterodimers [32]. Additionally, integrins can function as coreceptors for TLR2. Although membrane integrin α3β1 does not directly bind the lipopeptide ligand Pam3, blocking of the α3-subunit decreased TLR2-mediated IL-6 release from macrophages [33]. Furthermore, the functional association of other receptors such as C-X-C motif chemokine receptor 4 (CXCR4), a seven-transmembrane G-protein-coupled chemokine receptor, with TLR2 inside lipid rafts, led to a downregulation of the TLR2 response [34,35]. Moreover, proteins such as mannan-binding lectin (MBL), which modify TLR3 activation by interacting with poly(I:C), suppressed the poly(I:C)-induced activation of the TLR3 pathway and the subsequent cytokine production [36]. Additionally, several studies demonstrated that certain TLR ligand combinations induced a synergistic production of proinflammatory cytokines, which likely occur at the transcriptional level and involve the activation of multiple signaling pathways and transcription factor families [37,38,39,40,41]. Based on our present data, VEGF-R2 can be designated as an additional coreceptor for TLR2.

Many cytokines such as VEGF (or other basic proteins) can bind to membrane-bound heparan sulfate proteoglycans to become presented to their cognate signaling receptors such as VEGF-R2. However, this type of interaction appears not to be involved in the synergistic action of 18S rRNA/Pam2, since the pretreatment of macrophages with heparinase to remove cell membrane-localized heparan sulfate glycosaminoglycans and any associated proteins did not influence the 18S rRNA/Pam2-mediated cytokine induction. Al-though the mechanism for the coreceptor role of VEGF-R2 in 18S rRNA/Pam2-induced cytokine expression needs to be investigated in more detail, our results demonstrate that rRNA appears to induce interactions between VEGF-R2 and TLR2, which are necessary for the increased inflammatory response towards Pam2. These data are reminiscent of the previously characterized interaction between VEGF-R2 and its coreceptor neuropilin-1, which was reinforced by eRNA as well [6,22]. Accordingly, the expression of specific cofactors (e.g., CD36, CD14) can vary between different organs and cell types, which might explain the cell-type specificity of 18S rRNA/Pam2-mediated synergistic effects (https://www.proteinatlas.org/ENSG00000135218-CD36/tissue accessed on 27 January 2022). Ongoing studies are currently in progress to clarify these differences.

The synergistic influence of 18S rRNA on TLR2 activation also depends on the type of TLR2 ligand. While the release of cytokines induced by MALP-2, another TLR2/TLR6 activator, was also increased in the presence of 18S rRNA, the ligand concentration needed for this activation was much higher compared to Pam2 and comparable to the extent of activation for the TLR2/TLR1 ligand Pam3. These results indicate that rRNA might increase the binding of Pam2 to its receptor to a much higher degree in comparison to the other investigated agonists. This was confirmed by in-vitro binding assays, demonstrating that the binding affinity of the TLR2 ligand Pam2 to TLR2 was increased in the presence of 18S rRNA, which might be the reason for the observed higher inflammatory response in the cellular studies.

In accordance with our findings, it has been suggested that both the acyl groups as well as the N-terminal peptide moieties of the lipopeptide ligands are critical for their TLR2-dependent activating efficiency [30,42,43]. In this study, we were able to prove for the first time our hypothesis on the existence of a complex between 18S rRNA and Pam2 that is most likely based on the ionic interaction of positively charged amino acids in Pam2 and the negatively charged rRNA backbone. Therefore, the above-cited data could likewise depend on these structural features of rRNA. Yet, whether differences in fatty acid composition or other structural features of lipopeptides may influence the interactions between rRNA and TLR2 to promote the observed strong synergistic inflammatory response is currently under investigation.

The 18S rRNA secondary structure contains a high number of double-stranded regions and loops. To investigate whether the synergistic effect of 18S rRNA/Pam2 depends on sequence specificity and/or length, different fragments of 18S rRNA were generated. However, these 18S rRNA fragments did not show any significant differences in the expression of cytokines in the presence of Pam2. Consequently, the size, as well as the sequence of rRNA motifs, are likely not responsible for its synergistic influence, but it cannot be ruled out that secondary structural features of rRNA such as double-/single-stranded regions or stem-loops regions play a particular role. Therefore, the generation of 18S rRNA fragments with different secondary structures (hairpins, loops, etc.) will be investigated in future experiments.

We previously demonstrated, by blocking the TNF-α receptor, that longer periods of treatment of macrophages with eRNA/Pam2 included autocrine effects of TNF-α, resulting in an increased inflammatory potential [18]. It is well known that cellular interactions of TNF-α lead to the activation of NF-κB, and thereby increase not only its own expression and release at longer stimulation times but also that of other cytokines such as IL-6 [44,45]. Thus, to study only direct effects to elucidate the mechanism of activation of macrophages by 18S rRNA/Pam2 *in vitro*, only early time points were assessed. However, the release of cytokines, except for TNF-α, is too low after 2h of stimulation and, therefore, only the release of TNF-α was measured.

For *in vivo* experiments, we studied peritoneal cytokine production as an innate immune response towards the administration of the 18S rRNA/Pam2 complex. The model provides the opportunity to explore the effects of resident and recruited cells in the defined compartment of the peritoneum. In addition, for practical analytical reasons, a much larger lavage volume and higher cytokine concentrations in the peritoneum are an advantage compared to the respective analysis in blood. In fact, the preformed 18S rRNA/Pam2 complex was found to induce peritoneal cytokine production of TNF-α, IL-6, and MCP1, but not IL-1β *in vivo* in the peritoneum of mice, thereby corroborating the indicated cellular experiments. These data confirmed our previous *in vitro* findings, which demonstrated that IL-1β was not detectable in supernatants of macrophages even after 24 h of stimulation with eRNA/Pam2. Subsequently, activation of the inflammasome, which leads to the caspase-1-dependent release of Il-1β, seems not to be involved in synergistic activities of eRNA/Pam2 with the TLR2 activation [18,46]. However, it needs to be verified whether these complexes between eRNA and DAMPs/PAMPs can be formed *in vivo* as well, such as upon tissue damage or during infections. Several endogenous DAMPs such as HMGB1 were already shown to increase the sensitivity of PRRs and thereby promote the inflammatory response of viral or bacterial components [47,48]. Accordingly, it has been suggested that DAMPs, which accumulate during cell damage and aging, may play a role in the elevated severity and susceptibility of virus infections in the elderly [49]. Thus, the presence of small amounts of self eRNA released by processes of cell damage or during processes of sterile inflammation appears to sensitize the immune response by activating TLRs, induced by either a body’s DAMPs such as HMGB1 or by PAMPs during viral or bacterial load [9,14].

## 5. Conclusions

Taken together, our results indicate that the DAMP rRNA, released from damaged tissue under situations of sterile inflammations, serves as a strong cofactor in facilitating TLR2 ligand-induced proinflammatory activities. This sensitization reaction might favor the development of specific endogenous antagonists such as RNase1 to prevent hyperinflammatory reactions during processes of sterile inflammation or infectious diseases.

## Data Availability

The data presented in the current study are available on request from the corresponding author.

## Supplementary Materials

The following are available online at https://www.mdpi.com/article/10.3390/cells11091440/s1, Figure S1: Signaling pathways involved in 18S rRNA and Pam_2_ CSK_4_-induced cytokine expression; Figure S2: MALP-2-and Pam_3_ CSK_4_-induced *Tnf-α* mRNA expression; Figure S3: Pam_2_ CSK_4_-induced TNF-α and IL-6 expression in endothelial cells.
