# Supplementary material for: Extracellular Ribosomal RNA Acts Synergistically with Toll-like Receptor 2 Agonists to Promote Inflammation
Source: Cells. 2022 Apr 24;11(9):1440. doi: 10.3390/cells11091440 (PMC9103112; doi:10.3390/cells11091440)
Supplement: Supplementary file 1 [file cells-11-01440-s001.zip › cells-1595783-supplementary.pdf]

K. Grote et al.: Extracellular Ribosomal RNA acts synergistically with Toll-like Receptor 2 agonists to promote inflammation

Supplementary material

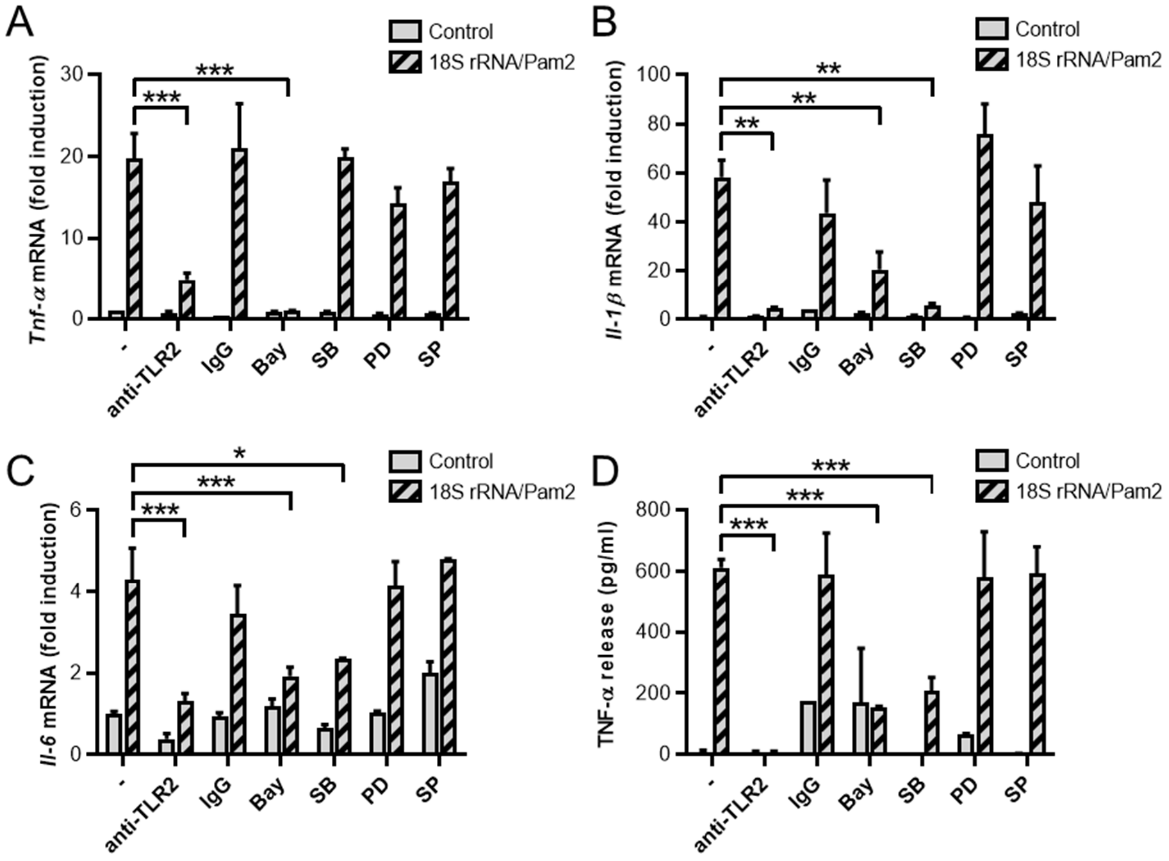

Figure S1. Signaling pathways involved in 18S rRNA and Pam$_2$CSK$_4$-induced cytokine expression. Macrophages (J774A.1 cells) were treated for 2 hours with preincubated mixture of Pam$_2$CSK$_4$ (Pam2, 0.1 ng/ml) together with 18S rRNA (1 µg/ml) after pretreatment of cells for 1h with neutralizing anti-TLR2 antibody (anti-TLR2, 2 µg/ml), or IgG control (IgG, 2 µg/ml), Bay 11-7082 (Bay, 5 µM), SB203580 (SB, 10 µM), PD98059 (PD, 20 µM), and SP 600125 (SP, 10 µM). Real-time PCR was used to determine transcript levels of *Tnf-α* (**A**), *Il-1β* (**B**), or *Il-6* (**C**). TNF-α protein levels in cellular supernatants were quantified by ELISA (**D**). PBS-treated cells without any additives served as control. Values are expressed as mean±SEM; N = 3-8; only significances between 18S rRNA/Pam2 group and inhibitor-treated groups of 18S rRNA/Pam2 groups were shown (*p<0.05, **p<0.01, ***p<0.001).

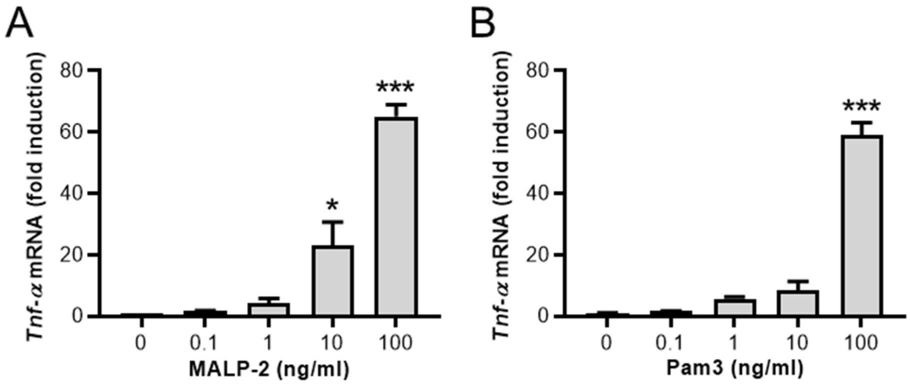

Figure S2. MALP-2- and Pam$_3$CSK$_4$-induced *Tnf-α* mRNA expression. Macrophages (J774A.1 cells) were treated for 2 h with different concentrations of MALP-2 (**A**) or Pam$_3$CSK$_4$ (Pam3) (**B**). PBS-treated cells without any additives served as control. Real-time PCR was used to determine transcript levels of *Tnf-α*. Values are expressed as mean±SEM; N = 3-5; *p<0.05, ***p<0.001 versus unstimulated control.

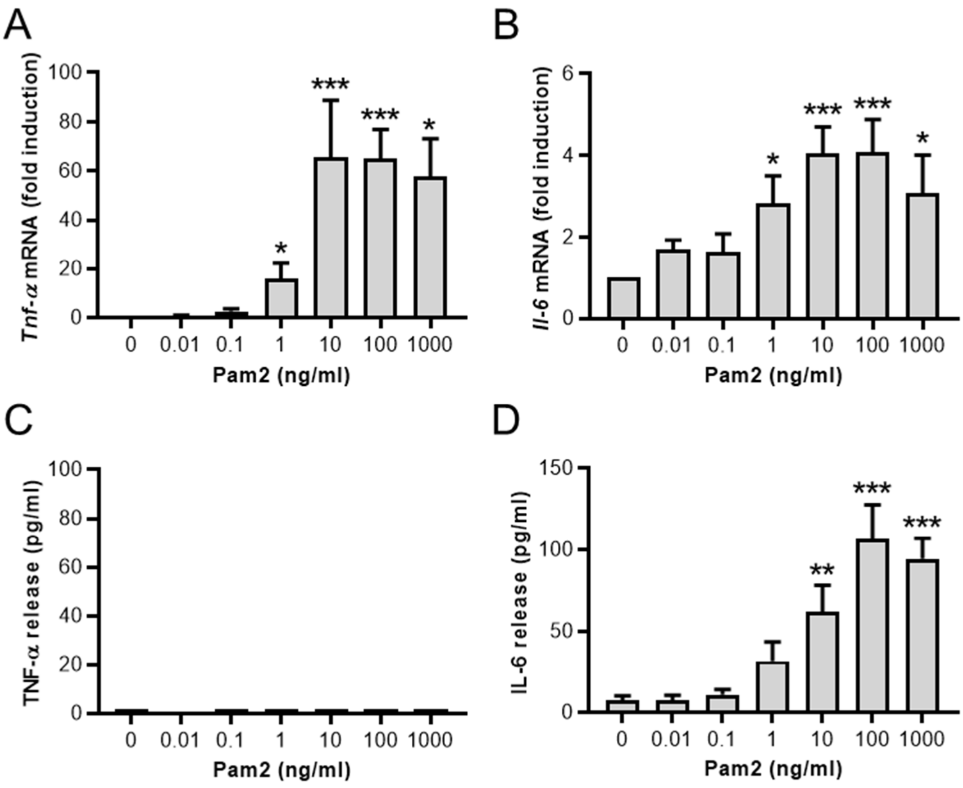

Figure S3. Pam$_2$CSK$_4$-induced TNF-α and IL-6 expression. Endothelial cells (MyEND cells) were treated for 3 h with different concentrations of Pam$_2$CSK$_4$ (Pam2). PBS-treated cells without any additives served as control. *Tnf-α* (**A**) and *IL-6* (**B**) mRNA levels were quantified by real-time PCR. The release of TNF-α (**C**) and IL-6 (**D**) protein levels in cellular supernatants was quantified by ELISA. Values are expressed as mean±SEM; N = 3-9; *p<0.05, **p<0.01, ***p<0.001 versus control value.
